# Homœopathy

**Published:** 1856-04

**Authors:** 


*Homœopathy.*—We have for a long time thought it unnecessary to attack the heresy of Hahnemann. The kind of mind likely to be attracted by it is unusually incapable of appreciating reasonable argument.

But now, in the decadence of the humbug, when it is growing shaky in the legs, and feeble in its hold upon the public confidence, we cannot avoid the ungenerous impulse to help it down the hill a little by a kick *a posteriori.*

Homœopathy, so called, has but little strength in our own city. The number of its practitioners is small, and they are not remarkable for talent of any sort. As we verily believe, not one of them is a true homœopath—at any rate, their patients are salivated with calomel, physicked with oz. doses of castor oil and salts and senna, put to sleep with grain doses of morphine (in one instance within our knowledge the sleep was eternal) and variously medicated in the manner of the regular profession. Of course all argument with such men is out of the question.

The subject has been brought to our mind by a perusal of the account of the death of the Czar Nicholas, of Russia. He was attended by a homœopathic physician. The cause of death assigned by this man was *paralysis pulmonum* and bronchitis. Paralysis of the lungs is a disease of rare occurrence. We have seen what we supposed to be it, following hooping-cough, and attended by atelectasis of the lung parenchyma. The voice was much changed, and it requires no argument to prove that it would, in an advanced state of the disease, be entirely lost. Yet we are told, in the official account of the death of the royal patient, that at the last he "took leave of his family with a firm voice"—a proceeding, says Mr. Wakley, of the London Lancet, "physically impossible if he was suffering from the diseases stated in the certificate of death." The bronchitis alone would have forbidden this.

We find this phrase "paralysis pulmonum" in nearly all our mortality reports, and it seems to be a fashionable cause for death among homœopaths and irregular German practitioners. It is hardly ever returned by educated or American physicians. *Palsy of the lungs* is a sounding designation to satisfy the questions of friends. This was doubtless the motive of the diagnosis in the case of the Czar Nicholas, but that it was not entirely satisfactory is proven by the sudden flight from St. Petersburg of the physician.

Touching the matter of homœopathic success in the treatment of disease, we give below a paragraph which we cut from the Boston Journal. It reminds us of a homœopath in Buffalo, who mentioned incidentally to the Health Physician that he had (in the summer of 1854) treated some thirty or forty cases of cholera without a death. An hour or two afterward he was waited upon by a health inspector, who politely requested him to report those cases, their names, ages, residence, &c., to him, under penalty of a fine of twenty-five dollars of each case. He was unable to do so, but the inspector, after listening to a confused argument about cases of cholera-morbus and diarrhœa, which *might* have been cholera, concluded that the fellow had really had no cases at all, and allowed him to escape the fines.

The extract below teaches very much the same lesson:

"*Homœopathic Treatment of Cholera.*—The following, which is extracted from the *Gazette des Hopitaux*, by the Montreal Medical Chronicle, is an interesting account of the results of homœopathic treatment in cholera, and comes from an authentic source.

"Dr. Bouquet, writing to the *Gazette des Hopitaax*, says: 'Homœopathy has just received a severe check in our town. You have, perhaps, heard of the noise it made last year with its pretended success in cholera. Dr. Chargé asserted that he had not lost one out of several hundred patients, and he published this statement in the political journals of Lyons and Bordeaux. When, during the present year, the scourge visited us anew, the authorities bestirred themselves, and thinking it was their duty to bring the truth to light, they entrusted one of the wards in Hotel Dieu, to Dr. Chargé. There, assisted by his colleagues in homœopathy, by pharmaciens, and by some young people his adepts, who devoted themselves to tending the patients (for he had found the ordinary staff insufficient and incompetent) he obtained the result which might easily have been anticipated; the broad day-light did not display sneccess. *Of twenty-six cholera patients admitted into this ward, twenty died, and Dr. Chargé withdrew.* To render the experiment conclusive, a ward had been set apart, in which the patients were treated by rational means, which did not profess to work wonders. *During the same period, of twenty-five patients admitted, but eleven died. Each ward had its turn of reception.* I think these facts are sufficiently decisive to render a renewal of such experiments needless, for if science profits by them, which is doubtful, humanity suffers not a little.'"

